# Structural studies of the unusual metal-ion site of the GH124 endoglucanase from *Ruminiclostridium thermocellum*

**DOI:** 10.1107/S2053230X18006842

**Published:** 2018-08-01

**Authors:** Saioa Urresti, Alan Cartmell, Feng Liu, Paul H. Walton, Gideon J. Davies

**Affiliations:** a York Structural Biology Laboratory, Department of Chemistry, University of York, York YO10 5DD, England; b Institute for Cell and Molecular Biosciences, Newcastle University, Newcastle upon Tyne NE1 7RU, England; c Department of Chemistry, University of British Columbia, Vancouver V6T 1Z1, Canada; d Department of Chemistry, University of York, York YO10 5DD, England

**Keywords:** endoglucanase, *Ruminiclostridium thermocellum*, *Clostridium*, metal ion, oligosaccharides, enzymes, carbohydrates, bio-inorganic, 2-oxohistidine

## Abstract

The recent discovery of ‘lytic’ polysaccharide monooxygenases, copper-dependent enzymes for biomass degradation, has provided new impetus for the analysis of unusual metal-ion sites in carbohydrate-active enzymes. In this context, the CAZY family GH124 endoglucanase from *Ruminiclostridium thermocellum* contains an unusual metal-ion site, which was originally modelled as a Ca^2+^ site but features aspartic acid, asparagine and two histidine imidazoles as coordinating residues, which are more consistent with a transition-metal binding environment. It was sought to analyse whether the GH124 metal-ion site might accommodate other metals. It is demonstrated through thermal unfolding experiments that this metal-ion site can accommodate a range of transition metals (Fe^2+^, Cu^2+^, Mn^2+^ and Ni^2+^), whilst the three-dimensional structure and mass spectrometry show that one of the histidines is partially covalently modified and is present as a 2-oxohistidine residue; a feature that is rarely observed but that is believed to be involved in an ‘off-switch’ to transition-metal binding. Atomic resolution (<1.1 Å) complexes define the metal-ion site and also reveal the binding of an unusual fructosylated oligosaccharide, which was presumably present as a contaminant in the cellohexaose used for crystallization. Although it has not been possible to detect a biological role for the unusual metal-ion site, this work highlights the need to study some of the many metal-ion sites in carbohydrate-active enzymes that have long been overlooked or previously mis-assigned.

## Abstract 2

The CAZY family GH124 endoglucanase from *Ruminiclostridium thermocellum* contains an unusual metal-ion site featuring a covalently modified ‘2-oxohistidine’ residue. Atomic resolution (∼1 Å) three-dimensional analyses, in conjunction with ITC and mass spectrometry, show that this metal site can accommodate a diverse array of transition metals, all of which confer a stabilizing effect on the enzyme.

## 1. Introduction   

**Figure 1:**
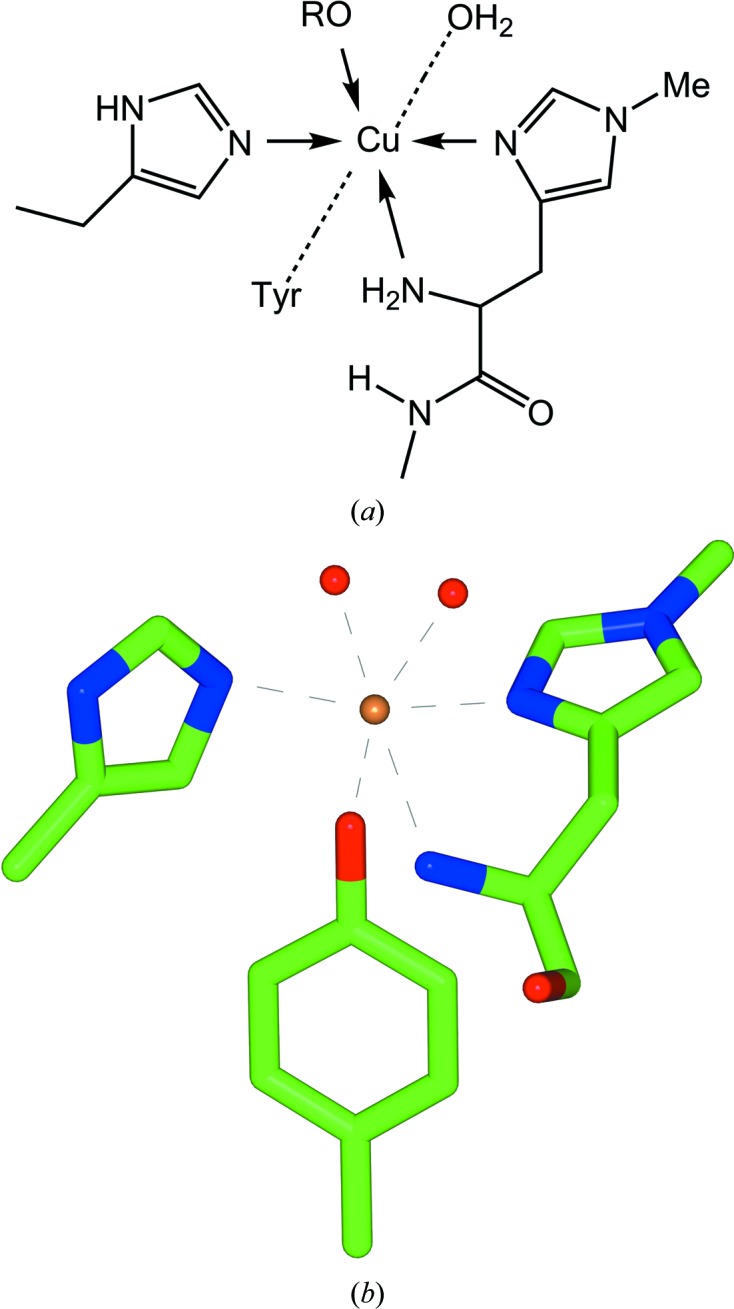
Model metal-ion site in LPMOs (PDB entry 2yet). (*a*) Schematic and (*b*) crystal structure of the copper-binding site from *Thermoascus aurantia­cus*, *Ta*AA9 (previously known as *Ta*GH61), including the typical histidine brace and a methylated His1 (Quinlan *et al.*, 2011 ▸).

Unarguably one of the most important recent developments in our understanding of the degradation of plant, and other recalcitrant, polysaccharides has been the discovery of (lytic) polysaccharide monooxygenases (LPMOs, sometimes termed PMOs). LPMOs are oxygenases that harness an unusual copper-containing ‘histidine-brace’ active centre to generate reactive oxygen species (from O_2_ or, under specific laboratory conditions, H_2_O_2_), ultimately leading to oxidative chain cleavage (reviewed, for example, in Horn *et al.*, 2012 ▸; Lo Leggio *et al.*, 2012 ▸; Span & Marletta, 2015 ▸; Walton & Davies, 2016 ▸). These enzymes are now classified in the Carbohydrate-Active enZymes (CAZy) classification as ‘auxiliary enzyme’ (Levasseur *et al.*, 2013 ▸) activities AA9, AA10, AA11, AA13, AA14 and AA15. The first structure of what is now known to be an AA10 LPMO (then believed to be a noncatalytic chitin-binding domain; Vaaje-Kolstad *et al.*, 2005 ▸) revealed an unusual metal-ion site featuring two histidine N atoms and the amino-terminal amine coordinating a metal that was modelled as an Na^+^ ion (PDB entry 2ben), thus not arousing the interest of the redox-enzyme community. Indeed, when it was shown that the function of these enzymes was the oxidative cleavage of polysaccharides harnessing molecular oxygen (Vaaje-Kolstad *et al.*, 2010 ▸), it was still claimed that any one of Na^+^, Ca^2+^ or Mg^2+^ was the potential metal ion at the active site. Other work on fungal AA9 enzymes had also implicated Ni^2+^, Mn^2+^ Co^2+^ or Mg^2+^ in their structures or mode of action, leading to considerable confusion in the field (Karkehabadi *et al.*, 2008 ▸; Harris *et al.*, 2010 ▸). It was only in 2011 that Quinlan and coworkers showed that the related AA9 enzymes were copper-dependent enzymes (Quinlan *et al.*, 2011 ▸), as subsequently confirmed by others (Phillips *et al.*, 2011 ▸), that the true metal ion for LPMOs became known (Fig. 1 ▸). A further finding of this latter study was that one of the coordinating residues in fungal AA9 enzymes is a covalently modified, methylated histidine (Quinlan *et al.*, 2011 ▸; Fig. 1 ▸).

Modelling of metal-ion sites in protein three-dimensional structures can be fraught with difficulty, and many metal-ion sites are both misinterpreted and potentially ‘contaminated’ with crystallization metal ions or metals used in affinity-purification steps (Schöneich, 2000 ▸; Zheng *et al.*, 2008 ▸, 2014 ▸; Chruszcz *et al.*, 2010 ▸). So, mindful of the potential of metal-ion sites to provide novel catalytic functions, of the environment of such sites to feature novel, modified amino acids, and of the possibility that such sites may have been overlooked in the original structural determinations, we were minded to examine the deposited three-dimensional structures for some represented enzyme families to look for such features.

Thus, whilst inspecting the PDB for unusual metal-ion sites, our attention was drawn to a metal site in the *Rumini­clostridium thermocellum* (the organism previously known as *Clostridium thermocellum*; Yutin & Galperin, 2013 ▸) GH124 endoglucanase GH124A. This enzyme is not part of the megadalton cellulosome complex of the organism. Instead, its modular structure consists of a cell-surface attachment ‘dockerin’ domain followed by a CAZY GH124 catalytic domain (which, in seminal work, Brás and coworkers showed to be an inverting endoglucanase; Brás *et al.*, 2011 ▸). This work also identified the active centre and revealed the first three-dimensional structure for the catalytic domain of a GH124 family member (reviewed in CAZypedia; The CAZypedia Consortium, 2018 ▸).

Initially modelled as a Ca^2+^ site in PDB entry 2xqo, we noticed that the geometry and coordination, featuring aspartic acid, asparagine and two histidine imidazoles, was more likely to indicate a transition-metal site. Furthermore, unusual unmodelled density at His157 led us to believe that this amino acid was also covalently modified, although in a manner different to that seen in LPMOs. We therefore sought to determine whether this metal-ion site could accommodate transition metals and how they might impact on stability, and to probe the unusual histidine modification.

Here, we show that the metal-ion site of GH124 can accommodate a variety of metal ions (but not Ca^2+^) and that the histidine is modified by a carbonyl group at C2 to make 2-oxohistidine (but only at a fraction of approximately 0.5). Metal binding also occurs with a stoichiometry of approximately 0.5 (in solution by ITC and in the crystal through occupancy refinement) and we are thus unable to deconvolute whether the 2-oxohistidine is required for metal binding or antithetical to it. In passing, we determined a series of structures of GH124 co-crystallized with cellohexaose which revealed the unusual binding of a contaminant, a terminally fructosylated cellooligosaccharide, with unambiguous density at ∼1 Å resolution. The work highlights the hidden world of metal-ion sites and unusual coordination geometries and encourages further study of these phenomena.

## 2. Materials and methods   

### 2.1. Macromolecule production   

The original *rtgh124*-containing plasmid was kindly provided by Professor Harry Gilbert’s laboratory (Brás *et al.*, 2011 ▸). This clone lacks the dockerin domain, but expresses 23 extra residues at the N-terminus, including a noncleavable His tag. The gene was subcloned using the ligation-independent cloning technique on a modified pET-28a plasmid containing a His tag (6×His) followed by a 3C protease recognition site at the N-terminus. Thus, the new clone was His-3C-*Rt*GH124 (see Table 1 ▸). The cleaved sequence has only three extra residues preceding the *Rt*GH124 protein (Gly-Pro-Ala). Furthermore, we have applied the UniProt numbering (A3DCJ4), which includes the first 130 amino acids of the dockerin domain. Therefore, the numbering of this construct is shifted from the sequence reported by Brás *et al.* (2011 ▸) by exactly 107 residues. Competent *Escherichia coli* BL21(DE3)pLysS cells were transformed by heat shock with the recombinant plasmid for protein production. The starter culture was incubated in 300 ml Luria–Bertani (LB) broth in the presence of 30 µg ml^−1^ kanamycin and 35 µg ml^−1^ chloram­phenicol at 37°C and 180 rev min^−1^ overnight. Using this as an inoculum, protein production was induced by the addition of 1 m*M* isopropyl β-d-1-thiogalactopyranoside (IPTG) to a total of 3 l of LB with the same antibiotics, temperature and shaking speed for 16 h. The cells were harvested by centrifugation at 5000*g* for 15 min. The pellets were resuspended in 100 ml buffer *A* (50 m*M* Tris pH 7.5, 300 m*M* NaCl, 20 m*M* imidazole) with the addition of one EDTA-free protease-inhibitor cocktail tablet (Roche). The cell suspension was then sonicated in an ice bath using five cycles of 60/90 s on/off pulses in a Soniprep 150 Plus sonicator (MSE). 50 ml of sample was incubated with 3 µl Benzonase (Santa Cruz Biotechnology) for 20 min at room temperature and then centrifuged (6000*g*, 20 min) and the supernatant filtered (45 µm). The filtrate was applied onto a 5 ml HisTrap column (GE Healthcare) pre-equilibrated in buffer *A*. The protein was eluted with a gradient to 500 m*M* imidazole in the same buffer and buffer-exchanged using a 30 kDa Vivaspin 20 filter unit (Sartorius). The low-salt and low-imidazole sample was incubated with a 1:20 ratio of His-tagged 3C protease for 18 h at 20°C at minimum rocking/rotating speed. The sample was then applied onto a second 5 ml HisTrap column (in the same buffer). The cleaved protein, pooled from the flowthrough, was then buffer-exchanged and concentrated using a 10 kDa Vivaspin 20 filter unit. A final volume of 2 ml protein in 50 m*M* Tris pH 7.5 was incubated with excess ethylenediaminetetraacetic acid (EDTA; 5 m*M*) for 18 h at 4°C with the aim of chelating any residual metal from the sample. Finally, the sample was run through a Superdex 75 16/60 gel-filtration column (GE Healthcare) previously equilibrated with demetallated buffer composed of 20 m*M* Tris pH 7.5, 150 m*M* NaCl. The eluted protein was pooled and concentrated using a 10 kDa Vivaspin 0.5 filter unit. The protein purity was confirmed by 12.5% SDS–PAGE.

### 2.2. Crystallization   

An optimization screen was designed for crystal growth, based on the conditions reported in Brás *et al.* (2011 ▸), as a gradient of PEG 3350 (16–26%) and Tacsimate buffer pH 5.0 (6–12%; Hampton Research). Either 2 m*M* metal salt [iron(II) chloride, manganese(II) acetate, nickel(II) chloride, calcium chloride or copper(II) chloride] or an equivalent volume of buffer (no metal) was added to each screen. Protein at 40 mg ml^−1^ was incubated with 6.7 m*M* of either cellotriose (G3; Megazyme) or cellohexaose (G6; Megazyme; this substrate turned out to be contaminated as revealed by three-dimensional analyses and will therefore be referred to as G5F, see below) in 20 m*M* Tris pH 7.5 for 18 h prior to crystal plate setup. Crystals grew at 20°C, mainly in clusters, after 3 d. Crystals of a reasonable size (∼100 nm) were harvested from several conditions and flash-cooled in liquid nitrogen after cryoprotection with a solution composed of 2 m*M* metal salt and concentrations of Tacsimate and PEG 3350 that were 2%(*v*/*v*) and 1%(*w*/*v*) higher, respectively, than those in the mother liquor (see Table 2 ▸). Two representative structures with the ligands G3 and G5F have been deposited with PDB codes 6g1g and 6g1i, respectively, and are used in the reports and figures in this paper.

β-1,4-linked Glc-Fru was synthesized following the conditions described previously in the literature (Hicks *et al.*, 1983 ▸). Glc-Glc-Fru was prepared by feeding Glc-Fru to an *Agrobacterium* sp. β-glucosidase-derived glycosynthase Abg2F6 (essentially as described by Kim *et al.*, 2004 ▸) in the presence of α-glucosyl fluoride (α-GlcF). Glc-Fru and Glc-Glc-Fru were included in co-crystallization, but no crystals grew under these conditions.

### 2.3. Data collection and processing   

Diffraction data (Table 3 ▸) were collected on beamline I02 at Diamond Light Source (DLS), Oxfordshire, England. In general, data sets were collected at a wavelength of 0.979 Å. X-ray fluorescence (XRF) spectra of the crystals were taken *in situ* during data collection to confirm the presence and nature of the metals in the cryoloop. Data sets were automatically indexed and integrated with *XDS* (Kabsch, 2010 ▸) and scaled with *AIMLESS *using *xia*2 (Winter, 2010 ▸) at DLS.

### 2.4. Structure solution and refinement   

XRF data were analysed with the *PyMCA* software (Solé *et al.*, 2007 ▸). Autoprocessed data sets were solved and refined using the *CCP*4*i*2 GUI (Potterton *et al.*, 2018 ▸; Table 4 ▸). The original structure reported by Brás *et al.* (2011 ▸) was initially truncated with *Sculptor* (Bunkóczi & Read, 2011 ▸) and used as a model for molecular replacement with *MOLREP* (Vagin & Teplyakov, 2010 ▸). Structures were refined with several cycles of *REFMAC*5 (Murshudov *et al.*, 2011 ▸) and *Coot* (Emsley *et al.*, 2010 ▸). Structures were generally validated using several tools in *Coot* and the European Protein Data Bank (PDBe; http://www.ebi.ac.uk/pdbe) validation tool. Sugars were valid­ated with *Privateer* (Agirre *et al.*, 2015 ▸) and metal correctness was tested with the *CheckMyMetal* (CMM) metal-binding site validation server (Zheng *et al.*, 2014 ▸, 2017 ▸). Structural figures were made with *CCP*4*mg* (McNicholas *et al.*, 2011 ▸).

### 2.5. Differential scanning fluorimetry   

Two replicas of each sample were prepared in thin-wall 0.2 ml tubes (Axygen) with 2 mg ml^−1^ (86 µ*M*) pure metal-free *Rt*GH124 protein in 50 m*M* Tris pH 7.5, 150 m*M* NaCl buffer (other buffers and pH values were also tried with similar results). A total of eight samples (plus replicas) were tested with protein only, 1 m*M* EDTA or one equivalent (86 µ*M*) of metal salt: iron(II) chloride, manganese(II) acetate, nickel(II) chloride, calcium chloride or copper(II) chloride. Samples (15 µl) were mixed with 1000× of the fluorescent dye SYPRO Orange (Sigma–Aldrich) in a 1:1 ratio (final volume 30 µl). Measurements were performed using an Agilent MX3000P QPCR machine. The temperature was increased by 1°C at 30 s intervals from 25 to 96°C and fluorescence was measured with excitation and emission wavelengths of 517 and 585 nm, respectively.

### 2.6. Electrospray (ESI-MS)   

The protein was tested by electrospray ionization mass spectrometry (ESI-MS) to observe the possible impact of metals on the His264 modification. Briefly, 200 µ*M* *Rt*GH124 samples were pre-incubated with 0.5 m*M* EDTA, 200 µ*M* metal salt [copper(II) chloride, iron(II) chloride, manganese(II) acetate or nickel(II) chloride] or no metal in pre-chelated 50 m*M* ammonium acetate pH 5.8 buffer. The experiment was conducted on a Waters LCT Premier XE system with the *MassLynx* 4.1 software. The system was calibrated with sodium formate solution and calibration was verified and corrected with horse heart myoglobin (16 951.5 ± 1.5 Da). Samples were diluted directly into acetonitrile–water–formic acid (50:50:0.1). Data were collected for 3 min over a scan range of 200–2000 *m*/*z*. The scans were combined and the baseline was subtracted. Data in the *m*/*z* range containing the peak series were then processed using the Maxent1 procedure over an appropriate mass range with suitable peak half-width settings determined from the raw data.

### 2.7. Proteolysis and mass spectrometry   

To empirically localize the oxidized residue, a peptide proteolysed from *Rt*GH124 was analysed by liquid chromatography and tandem mass spectrometry (LC-MS/MS). 25 µg *Rt*GH124 was run on a 12.5% SDS–PAGE gel. The gel was stained in a clean box with SimplyBlue SafeStain (Invitrogen) and the appropriate band was cut. In-gel sequential digestion with trypsin and Asp-N endoproteases was performed after reduction with dithioerythritol and *S*-carbamidomethylation with iodoacetamide. The peptide mixture was loaded onto a nanoAcquity UPLC system (Waters) equipped with a nanoAcquity Symmetry C18 5 µm trap column (180 µm × 20 mm, Waters) and a nanoAcquity HSS T3 1.8 µm C18 capillary column (75 µm × 250 mm, Waters). The trap wash solvent was 0.1%(*v*/*v*) aqueous formic acid and the trapping flow rate was 10 µl min^−1^. The trap was washed for 5 min before switching the flow to the capillary column. Separation used a gradient elution of two solvents [solvent *A*, aqueous 0.1%(*v*/*v*) formic acid; solvent *B*, acetonitrile containing 0.1%(*v*/*v*) formic acid]. The capillary column flow rate was 350 nl min^−1^ and the column temperature was 60°C (unlike similar columns, we were able to run nanoAcquity HSS T3 1.8 µm C18 capillary columns at 60°C, which improves mass-transfer rates and thus resolution and also reduces back pressure in the system, enabling a slight increase in flow rate). The gradient profile was a linear 2–35% solvent *B* over 20 min. All runs then proceeded to a wash with 95% solvent *B* for 2.5 min. The column was returned to the initial conditions and re-equilibrated for 25 min before subsequent injections. The nanoLC system was interfaced with a maXis HD LC-MS/MS system (Bruker Daltonics) with a CaptiveSpray ionization source (Bruker Daltonics). Positive ESI-MS and MS/MS spectra were acquired using AutoMSMS mode. Instrument control, data acquisition and processing were performed using the *Compass* 1.7 software (*microTOF Control*, *Hystar* and *DataAnalysis*, Bruker Daltonics). The collision-energy and isolation-width settings were automatically calculated using the AutoMSMS fragmentation table, with an absolute threshold of 200 counts, preferred charge states 2–4 and singly charged ions excluded. A single MS/MS spectrum was acquired for each precursor and former target ions were excluded for 0.8 min unless the precursor intensity increased fourfold. Tandem mass spectra were searched against an in-house database containing the expected protein sequence (842 sequences; 329 761 residues) using a locally running copy of the *Mascot* program (v.2.5.1; Matrix Science) through the Bruker *ProteinScape* interface (v.2.1). The results were filtered to accept only peptides with an expect score of 0.05 or lower. Identification of oxidized histidine was confirmed by the manual validation of product-ion spectra against predicted b- and y-sequence ions.

### 2.8. Isothermal titration calorimetry   

380 µl of pure pre-chelated *Rt*GH124 protein (60 µ*M*) was titrated with 600 µ*M* metal salt [copper(II) chloride, iron(II) chloride, manganese(II) acetate or nickel(II) chloride] in 1 µl injections at 180 s intervals. The buffer used in both cell and syringe samples was 50 m*M* sodium acetate pH 5.0. Titrations were performed on a MicroCal Auto-ITC_200_ (GE Healthcare) at 25°C and 750 rev min^−1^ stirring speed. Data were analysed with the *Origin* 7 software (OriginLab) and blanks were subtracted from the original data.

### 2.9. Glucose- and fructose-detection methods   

Commercial sugars were detected by high-pressure anion-exchange chromatography (HPAEC) with pulsed amperometric detection and were separated on a Carbopac PA1 guard and analytical column. Isocratic elution with 20 m*M* sodium hydroxide for 30 min and then with a 60% linear gradient of sodium acetate in 100 m*M* sodium hydroxide over 60 min was used to separate the sugars. Sugars were detected using the carbohydrate standard quad waveform for electrochemical detection at a gold working electrode with an Ag/AgCl pH reference electrode. The glucose/mannose/fructose detection kit from Megazyme was also utilized to detect the relative amounts of glucose and fructose. This is a linked assay kit, with the detection of the appropriate monosaccharide linked 1:1 to the detection of NADH at *A*_340 nm_ using an extinction coefficient of 6230 *M* ^−1^ cm^−1^.

## 3. Results and discussion   

### 3.1. Effect of the metal on thermal stability   

**Figure 2:**
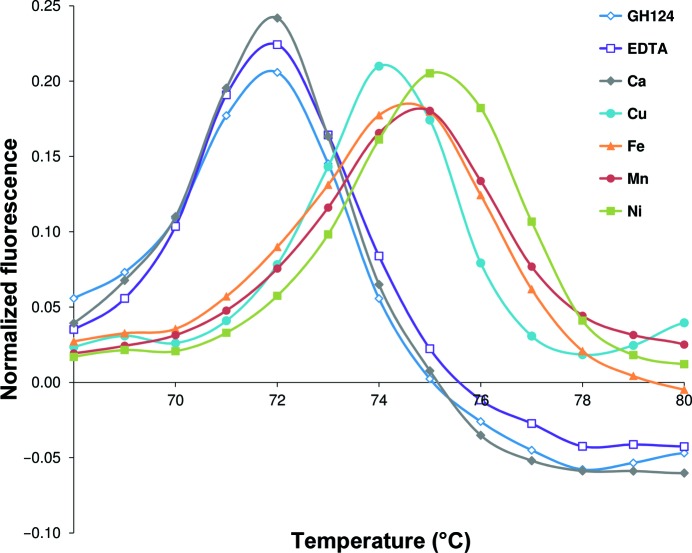
Differential scanning fluorimetry is represented as normalized fluorescence as a function of temperature. Controls with protein only (GH124) and EDTA are compared with samples containing one equivalent of each metal, *i.e.* calcium, copper, iron, manganese or nickel. Both controls and the sample with calcium show a maximum in fluorescence at ∼72°C. The melting temperature is shifted to 74–75°C in the presence of transition metals.

In order to provide a preliminary indication of metal binding, the thermal stability of *Rt*GH124 was studied by differential scanning fluorimetry (Fig. 2 ▸). Both controls (protein and protein + EDTA) show a melting transition at 72°C. Addition of Ca^2+^ did not shift the melting temperature, suggesting that this metal may not bind the protein, at any pH between pH 5 and 7.5, and thus hints that the assignment of this metal in the original deposition may be misleading. In contrast, the melting temperature of *Rt*GH124 was shifted by +2–3°C when transition metals such as copper, nickel, manganese or iron were tested. Similar behaviour has also been replicated in other buffers and at other pH values (data not shown).

### 3.2. Isothermal titration calorimetry   

**Figure 3:**
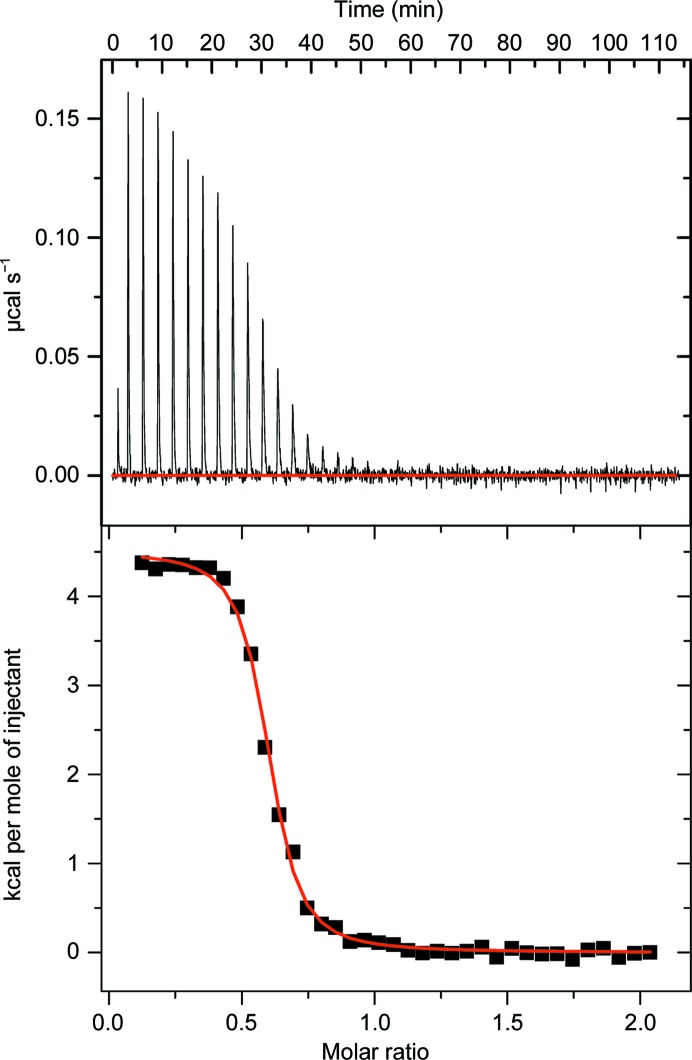
Representative ITC experiment: titration of manganese(II) acetate with *Rt*GH124. The upper panel depicts raw binding heats and the baseline (in red). The lower panel shows corrected integrated heats and the fit to a single-site binding model (red line).

To investigate further the interaction between *Rt*GH124 and metals, ITC experiments were carried out. The manganese binding isotherm at pH 5, as representative of the other metals tested, is depicted in Fig. 3 ▸. The reactions were exothermic (with the exception of Cu^2+^ which was endothermic) and Ca^2+^ showed no binding (over the range tested from pH 5 to pH 7; see Table 5 ▸), in accord with the fluorimetry results. The low stoichiometries (*N* ≃ 0.5) on metal binding may reflect partial modification of the metal-coordinating histidine (discussed below).

### 3.3. Three-dimensional structure of *Rt*GH124   

**Figure 4:**
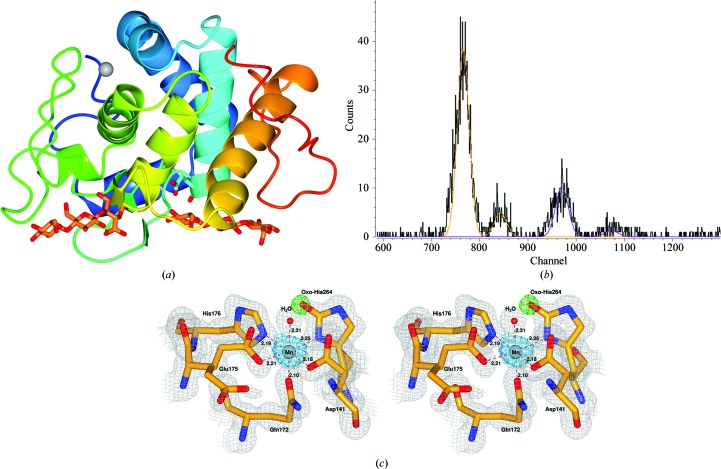
Three-dimensional structure of *Rt*GH124 and metal-ion site. (*a*) General rainbow view of *Rt*GH124 (PDB entry 6g1g) from red (N-terminus) to blue (C-­terminus) in complex with two co-crystallized cellotrioses (in orange) next to the catalytic Glu203 (in light blue) and manganese (grey sphere) at the metal-ion site. (*b*) X-ray fluorescence spectrum for PDB entry 6g1g. Peaks corresponding to manganese and nickel are shown as yellow and purple lines, respectively. (*c*) Stereoview of the metal-ion site. The 2*F*_o_ − *F*_c_ electron-density map (grey mesh) is contoured at 1σ (0.44 e Å^−3^). The *F*_o_ − *F*_c_ difference OMIT map (green mesh), calculated without the 2-oxo modification in His264, is contoured at 3σ (0.36 e Å^−3^). The anomalous map (blue mesh) is likewise contoured at 3σ (0.36 e Å^−3^). Distances between the metal and the coordinating atoms of Asp141, Gln172, Glu175, His176, His264 and the water are shown as red dashed lines.

As originally described in the seminal publication by Brás *et al.* (2011 ▸), *Rt*GH124 is composed of seven α-helices surrounding an eighth (Fig. 4 ▸ *a*) which contains the catalytic acid Glu203 (Glu96 in Brás *et al.*, 2011 ▸). The metal-ion site (Fig. 4 ▸ *c*) is located far from the catalytic area, and thus no impact has been observed on hydrolytic activity in the presence of different metals (data not shown). Manganese is coordinated by Asp141, Gln172, Glu175, His176, His264 and a water molecule. In several structures with other metals, the water is at a very low occupancy or is absent, while Glu175 is often found in a double conformation, with only one of its positions coordinating the metal. The reasons for this are unclear: it may reflect pH (5–6), the partial metal occupancy or histidine modification. As mentioned above, high-resolution structures of *Rt*GH124 (1.40–0.94 Å) have revealed an unambiguous modification of C2 of His264 (His157 in Brás *et al.*, 2011 ▸). In the case of manganese, the metal and the His264 modification both display partial occupancies of 0.7 and 0.3, respectively. Occupancy values were similar for other metals under similar conditions, varying from 0 to 0.5 for the histidine modification and from 0 to 0.8 for the metal. X-ray fluorescence spectra proved to be a powerful, but seldom conclusive, tool: several metals were usually found in each sample, along with the one added in the crystallization condition, with nickel appearing in all of the spectra as a common metal. These contaminants are likely to be derived from the purification steps or buffers used for the crystallization conditions. In the case of manganese this appeared to be the main metal in the sample, with only some residual nickel (Fig. 4 ▸ *b*). It is worth noting that this technique reveals which metals are present in the cryoloop and not solely what is present in the metal-ion site.

### 3.4. Mass spectrometry of the covalent modification   

**Figure 5:**
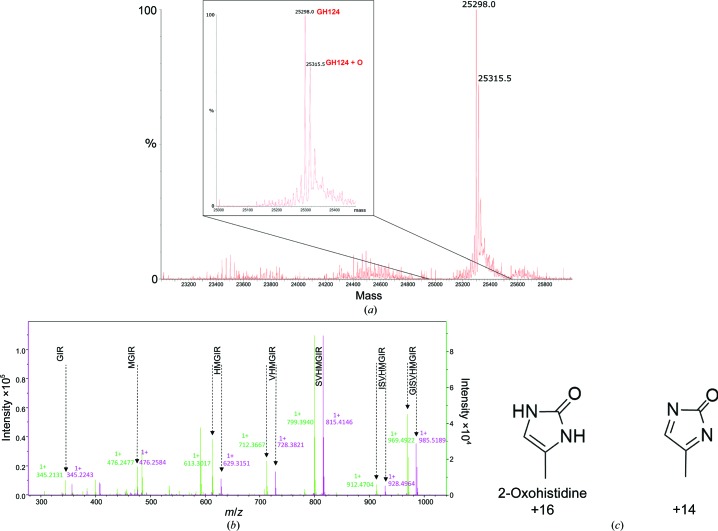
Mass spectrometry was performed on cleaved (non-His-tagged) *Rt*GH124 to study the His264 modification. (*a*) ESI-MS, general and enlarged (inset) spectra. The two main peaks indicate a mixture of non-oxidized (calculated, 25 304 Da; observed, 25 298 Da) and oxidized (calculated, 25 320 Da; observed, 25 315 Da) forms. The difference in the observed and calculated sizes is within the error range for this technique. (*b*) The peptide resulting from the proteolysis of cleaved *Rt*GH124 (GISV**H**MGIR) was analysed by LC-MS/MS. A mixture of unoxidized (in green) and oxidized (in pink) forms was also observed as a shift of 16 Da in the peaks of peptides containing His264. (*c*) The form of ‘2-oxohistidine’ observed here is that which gives the +16 mass shift [as described in Schöneich (2000 ▸) and observed in the *B. subtilis* PerR structure (Traoré *et al.*, 2009 ▸)] and not the chemically distinct +14 species.

To assess the nature of the histidine modification, mass-spectrometric assays were carried out. ESI-MS (Fig. 5 ▸ *a*) clearly shows two main peaks corresponding to a mixture of modified and unmodified protein. The difference between the peaks is 16 Da, corresponding to the extra O atom in the modified species. To confirm that the modification was indeed in His264, LC-MS/MS analysis was performed after *Rt*GH124 proteolysis (Fig. 5 ▸ *b*). This technique provided unequivocal evidence of the modification taking place in His264, observed as a gap of 16 Da between each pair of peaks (unoxidized and oxidized forms) for peptides containing this residue (**H**MGIR to GISV**H**MGIR). Thus, the combination of crystallographic and MS techniques allowed it to be determined that this modified residue 264 is indeed 2-oxohistidine [note that what is called ‘2-oxohistidine’, PDB residue ID OHI, reflects two chemically different species which would give rise to mass shifts of +14 or +16; what is observed here is the +16 species (Fig. 5 ▸ *c*) as described in Schöneich (2000 ▸) and observed in the *Bacillus subtilis* PerR structure (Traoré *et al.*, 2009 ▸)]. The fact that the oxidation is only partial could be a factor affecting the ITC stoichiometry, especially in light of the loss of metal binding by PerR upon oxidation (at one of its two 2-oxohistidine residues).

Amino-acid modification, and histidine oxidation in particular, is a well known feature in nature (Schöneich, 2006 ▸). Histidine has been extensively reported as being one of the most susceptible amino acids to free-radical reactions mediated by (transition) metals, mainly when they are in close proximity (Uchida & Kawakishi, 1993 ▸; Schöneich, 2000 ▸; Hovorka *et al.*, 2002 ▸; Lam *et al.*, 2010 ▸). The modified 2-oxihistidine has been reported as an oxidative stress marker both in aerobic and (facultative) anaerobic microorganisms (Uchida & Kawakishi, 1993 ▸; Ahn & Baker, 2016 ▸; Sethu *et al.*, 2016 ▸). In PerR, the protein functions as a metal-dependent H_2_O_2_ sensor.

### 3.5. Observation of a fructosylated oligosaccharide   

**Figure 6:**
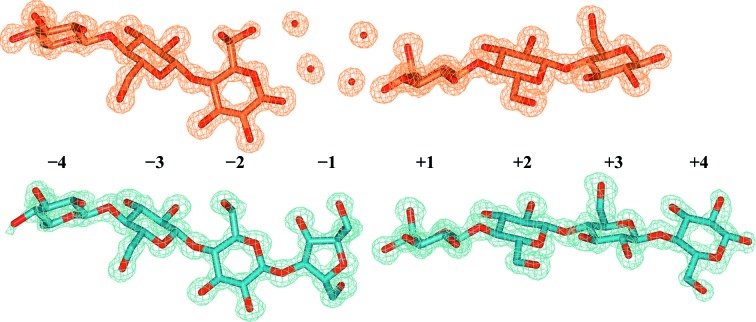
Comparison of the sugars found in the catalytic site. When co-crystallized with G3 (above, orange), two cellotrioses were found on either side of the catalytic residue, with the chains separated by several water molecules. When co-crystallized with the G5F-contaminated cellohexaose substrate (below, blue) no cleavage was found. Furthermore, the fructose unit substituted the waters that were very well defined in the previous structure.

Structures of *Rt*GH124 co-crystallized with cellotriose (G3) showed an active site with two cellotrioses and two water molecules hydrogen-bonded to either O1 of glucose in the first chain or O4 of glucose in the second chain (Fig. 6 ▸, in orange), akin to the structure reported by Brás *et al.* (2011 ▸). In structures co-crystallized with our original batch of cellohexaose a very unusual and unexpected fructosylated oligosaccharide (which we term G5F) was observed in the active site (Fig. 6 ▸ *b*, blue).

Clear electron density indicated that the reducing-end sugar was not glucose, as expected, but a 1–4-linked fructose moiety. The hexasaccharide appears not to be cleaved, as four to six molecules of each chain have been observed in all of the structures (data not shown). Enzyme subsites −4 to −1 are occupied by one molecule and +1 to +4 by another, with both of them stabilized in the crystal by a second molecule in the same asymmetric unit.

Our initial thought was that the fructosylated oligosaccharide would be likely to represent a very minor contaminant in the G6 preparation, one somehow selected by the enzyme owing to tighter binding and/or inhibitory potential. We therefore speculated that it must bind very tightly and thus that fructosylated oligosaccharides might represent a new class of glycosidase inhibitor. With this in mind, two new β-1,4 ligands were synthesized to test as putative GH124 inhibitors: Glc-Fru (GF) and Glc-Glc-Fru (GGF). However, no crystals could be obtained by co-crystallization with either of these ligands, and no binding was detectable using ITC.

Given the apparent lack of binding of GGF to *Rt*GH124, we sought to test whether our assumption that the G5F is a tight-binding, but extremely minor, contaminant was correct. Digestion of cellohexaose samples, both those suspected of containing G5F and a newly purchased batch (believed to be fructose-free), was performed using a GH1 β-glucosidase from *Thermotoga maritima*. The monosaccharide content was detected by both high-performance anion-exchange chromatography (HPAEC) and the glucose/mannose/fructose detection kit from Megazyme. The originally purchased G6 sample contained an approximately 1:1 ratio of G6 and G5F (but subsequently purchased batches were 100% G6), highlighting that the unusual observed species was simply a contaminant in the commercial supply of G6 available at that time.

## 4. Conclusions   

*Rt*GH124 is the defining member of CAZY family GH124. The original publication highlighted its catalytic activity as an unusual inverting cellulose. Unusually for *R. thermocellum*, *Rt*GH124A is not targeted to the multi-enzyme cellulosome complex of the organism but instead consists of a ‘dockerin’ domain which instead targets the enzyme to the cell surface of the bacterium, followed by a CAZY GH124 catalytic domain; *Rt*Gh124 is therefore an unusual enzyme if its primary role is in cellulose degradation yet it is not part of the cellulose complex. The presence of a partial occupied covalently modified histidine in a site that binds a variety of transition metals is therefore intriguing. Histidine 2-oxidation is normally associated with oxidative stress response elements (see, for example, Traoré *et al.*, 2009 ▸) and has not, to our knowledge, previously been witnessed on-enzyme. Mass spectrometry shows it is present in the protein as expressed and is not a feature of X-ray exposure of the crystal. Whether it plays a role in stress signalling in *R. thermocellum* is solely a matter for conjecture, but it serves to highlight both the difficulty in assigning metal-ion sites in proteins and the potential for more, unusual, metal-ion sites that have gone unnoticed; most notably in the (L)PMO enzymes where the metal ion and histidine modification hid in plain sight for many years.

## Supplementary Material

PDB reference: GH124 endoglucanase, complex with G3, 6g1g

PDB reference: complex with G5F, 6g1i

## Figures and Tables

**Table 1:** Macromolecule-production information

Source organism	*R. thermocellum*
DNA source	Brás *et al.* (2011 ▸)
Forward primer	CTGTTCCAGGGACCAGCACCTGCAAATACACAATCC
Reverse primer	GGAGATTTATTACTAATCACATATGGCTAGC
Cloning vector	pET-28a
Expression vector	Modified pET-28a containing 3C protease site
Expression host	*E. coli* BL21(DE3)pLysS
Complete amino-acid sequence of the construct produced†	**HHHHHH**SSGLEVLFQGPAPANTQSGILNDGYFPPGTSKHELIARASSLKVSEVKAIIKKQVDEHWDVIRDVCGFKNKEVAYAFFFGMATRESTFRAATETGSGASHAFGPLQTAETAYANANPNYMPEHNVPEMHQYDFTEYNFYDVGISVHMGIRHFLHFARLAKEKYSGRDIARHGLMGYNTGWIDGADESWIVRYADETAALGAWYLRNNHMSDDEFTWDTDPRVDRSNPWEIYY

† The histidine tag is in bold. The 3C protease recognition site is underlined (cleavage occurs between Q and G). After 3C cleavage, the residues GPA remain at the N-terminus, preceding the *Rt*GH124 sequence.

**Table 2:** Crystallization

	*Rt*GH124–G3–Mn (PDB entry 6g1g)	*Rt*GH124–G5F–Mn (PDB entry 6g1i)
Method	Vapour diffusion, sitting drop	Vapour diffusion, sitting drop
Plate type	96-well 2-drop MRC	96-well 2-drop MRC
Temperature (K)	293	293
Protein concentration (mg ml^−1^)	40	40
Volume and ratio of drop	54 µl, 1:1 ratio	54 µl , 1:1 ratio
Volume of reservoir (nl)	400	400
Buffer composition of protein solution	20 m*M* Tris pH 7.5, 6.7 m*M* G3	20 m*M* Tris pH 7.5, 6.7 m*M* G5F
Composition of reservoir solution	12% Tacsimate pH 5.0, 18% PEG 3350, 2 m*M* manganese(II) acetate	6% Tacsimate pH 5.0, 20% PEG 3350, 2 m*M* manganese(II) acetate
Cryoprotection buffer solution	14% Tacsimate pH 5.0, 19% PEG 3350, 2 m*M* manganese(II) acetate	8% Tacsimate pH 5.0, 21% PEG 3350, 2 m*M* manganese(II) acetate

**Table 3:** Data collection and processing Values in parentheses are for the outer shell.

	*Rt*GH124–G3–Mn (PDB entry 6g1g)	*Rt*GH124–G5F–Mn (PDB entry 6g1i)
Diffraction source	I02, DLS	I02, DLS
Wavelength (Å)	0.98	0.98
Temperature (K)	100	100
Detector	PILATUS 6 M-F	PILATUS 6 M-F
Rotation range per image (°)	0.10	0.10
Total rotation range (°)	180	180
Exposure time per image (s)	0.04	0.04
Space group	*P*3_2_21	*P*2_1_2_1_2_1_
*a*, *b*, *c* (Å)	73.92, 73.92, 74.94	74.14, 74.14, 77.33
α, β, γ (°)	90, 90, 120	90, 90, 90
Mosaicity (°)	0.14	0.25
Resolution range (Å)	74.92–1.03 (1.06–1.03)	74.17–0.99 (1.01–0.99)
Total No. of reflections	1021012 (29079)	1203487 (15629)
No. of unique reflections	113462 (5523)	215828 (6811)
Completeness (%)	99.9 (98.7)	95.3 (62.0)
Multiplicity	9.0 (5.3)	5.6 (2.3)
〈*I*/σ(*I*)〉	15.9 (1.5)	12.6 (1.0)
*R*_p.i.m._	0.02 (0.44)	0.02 (0.63)
CC_1/2_	0.9 (0.6)	0.9 (0.5)
Overall *B* factor from Wilson plot (Å^2^)	10	7

**Table 4:** Structure solution and refinement

	*Rt*GH124–G3–Mn (PDB entry 6g1g)	*Rt*GH124–G5F–Mn (PDB entry 6g1i)
Resolution range (Å)	64.10–1.04	53.59–0.99
Completeness (%)	99.9	95.13
σ Cutoff	None	None
No. of reflections, working set	107700	204772
No. of reflections, test set	5714	10962
Final *R*_cryst_	0.13	0.13
Final *R*_free_	0.14	0.15
No. of non-H atoms		
Protein	1817	3589
Ion	1	2
Ligand	80	180
Water	270	475
Total	2168	4246
R.m.s. deviations		
Bonds (Å)	0.020	0.020
Angles (°)	1.9	1.9
Average *B* factors (Å^2^)		
Protein	15	11
Ion	13	9
Ligand	12	13
Water	30	24
Ramachandran plot		
Most favoured (%)	203	402
Allowed (%)	3	10
Outliers	1	0

**Table 5:** Isothermal titration calorimetry values at pH 5.0

	*N*	*K*_d_ (µ*M*)	Δ*H* (cal mol^−1^)	Δ*S* (cal mol^−1^ K^−1^)
Mn	0.8	386	3.77	15.6
Fe	0.5	37	4.05	20.3
Ni	0.5	621	1.52	14.7
Cu	0.5	7	−4.34	23.6
